# Antibacterial mechanism of fraxetin against *Staphylococcus aureus*

**DOI:** 10.3892/mmr.2014.2529

**Published:** 2014-09-02

**Authors:** HAITING WANG, DAN ZOU, KUNPEING XIE, MINGJIE XIE

**Affiliations:** Liaoning Provincial Key Laboratory of Biotechnology and Drug Discovery, College of Life Science, Liaoning Normal University, Dalian, Liaoning 116000, P.R. China

**Keywords:** fraxetin, cortex fraxini, *Fraxinus chinensis* Roxb, antibacterial mechanism, topoisomerase

## Abstract

Fraxetin is one of the main constituents of the traditional medicinal plant *Fraxinus rhynchophylla*. The inhibitory effect of fraxetin on various bacterial strains has been extensively reported, however, its mechanism of action on bacterial cells remains to be elucidated. In the present study, the antibacterial mechanism of fraxetin on *Staphylococcus aureus* was systematically investigated by examining its effect on cell membranes, protein synthesis, nucleic acid content and topoisomerase activity. The results indicated that fraxetin increased the permeability of the cell membrane but did not render it permeable to macromolecules, such as DNA and RNA. Additionally, the quantity of protein, DNA and RNA decreased to 55.74, 33.86 and 48.96%, respectively following treatment with fraxetin for 16 h. The activity of topoisomerase I and topoisomerase II were also markedly inhibited as fraxetin concentration increased. The result of the ultraviolet-visible spectrophotometry demonstrated that the DNA characteristics exhibited a blue shift and hypochromic effect following treatment with fraxetin. These results indicated that fraxetin had a marked inhibitory effect on *S.aureus* proliferation. Further mechanistic studies showed that fraxetin could disrupt nucleic acid and protein synthesis by preventing topoisomerase from binding to DNA.

## Introduction

In recent years, there has been a consistent increase in the number of antibiotic-resistant bacteria due to the extensive use of antimicrobial agents in domestic animals, which are subsequently transmitted to humans through the food chain. The virulence and pathogenicity of a bacterium increases with the increase in its antibiotic resistance. Therefore, it is important to identify alternative drugs that can replace traditional antibiotics, thereby reducing the development and spread of resistance. Additionally, elucidaqting the mechanism of action of these alternative compounds and the resistance of bacterium to these compounds provides essential information for basic microbiological research.

Fraxetin, a major constituent of the traditional medicinal plant *Fraxinus rhynchophylla*, has been found to possess multiple bioactivities, including scavenging reactive oxygen species and inhibiting lipid peroxidation in the rat brain (1,2). Previous studies have demonstrated that fraxetin has antibacterial activities against *Staphylococcus aureus*, however, its inhibitory mechanism remains to be elucidated (3–5). Fraxetin is widely available and relatively cheap, and is known to have few side effects, and low resistance as well as other beneficial properties. In the present study, *S. aureus* was used and the antibacterial mechanism of fraxetin was examined through studies on the permeability of the cell membrane and changes in the content of nucleic acid and soluble proteins in order to provide a theoretical basis for the development of antibacterial drugs with high efficacy and low toxicity.

## Materials and methods

### Materials

*S. aureus* (ATCC26112) was obtained from the Chinese Medicine Bacterial Preservation Centre (Beijing, China). Fraxetin, at 99% purity, was purchased from Nuowei Xin (Dalian, China). The fraxetin was dissolved in absolute ethanol and concentrated solutions were added to the bacterial cultures to maintain the lowest possible concentration of ethanol in the cultures. The restriction enzyme, pBR322, was purchased from Takara Bio, Inc. (Shiga, Japan). DAPI was purchased from Beyotime Institute of Biotechnology (Shanghai, China). Common chemicals (ethanol, NaCl, KCl, KH_2_PO_4_, K_2_HPO_4_, beef extract, peptone) were purchased from Tianjin Kemiou Chemical Reagent Co, Ltd. (Tianjin, China). SDS, Tris-base, bovine serum albumin, adenosine triphosphate and proteinase K were purchased from Sangon Biotech Co., Ltd. (Shanghai, China). All assays were performed according to the manufacturer’s instructions.

### Determination of the electrical conductivity of the culture medium

*S. aureus* was cultured to logarithmic phase, subpackaged in a test tube and treated with 0.05 mg/ml fraxetin at 37°C for 0, 1, 2, 3, 4, 5, 6, 7 and 8 h. The electrical conductivity of the culture medium was then determined, with ethanol used as a control group. Each experiment was repeated three times (6).

### Measurement of the quantity of DNA and RNA

*S. aureus* was obtained by centrifugation (3,000 × g for 10 min) and washed twice with phosphate-buffered saline (PBS; 135 mM NaCl, 2.7 mM KCl, 1.5 mM KH_2_PO_4_ and 8 mM K_2_HPO_4_; pH 7.0). The cells were suspended in PBS and treated with 0.05 mg/ml fraxetin for 0, 1, 2, 3, 4, 5, 6, 7 and 8 h. Following the reaction, the supernatant fluid was measured by ultraviolet-visible spectrophotometry (UV1100 model; Shanghai Tianmei Scientific Instruments Co., Ltd., Shanghai, China) to analyze the content of DNA and RNA, with ethanol used as a control group. Each experiment was repeated three times (7).

### Determination of the S. aureus soluble protein content

*S. aureus* was inoculated into 50 ml beef extract peptone medium (containing 3 g beef extract, 10 g peptone, 5 g NaCl and 1 litre distilled water; pH 7.0) with fraxetin (final concentration 0.05 mg/ml, with ethanol as a control group) and cultured in a rotary shaker (120 rpm) at 37°C for 16 h. Cells were collected and 0.5 mg of cells were suspended in 40 μl double evaporated water and 160 μl loading buffer, incubated in boiling water for 8 min and centrifuged to obtain supernatant. The supernatant was loaded in SDS-PAGE to quantitatively analyze the change in soluble protein content in *S. aureus* with ethanol as a control group.

### Determination of the DNA and RNA content of S. aureus

*S*. *aureus* was inoculated into beef extract-peptone medium with fraxetin (final concentration 0.05 mg/ml, ethanol as a control) and cultured for 12, 16, 20 and 24 h. The cells were then collected and resuspended in aquae sterilisata. Triple volume of 4′,6-diamidino-2-phenylindole (DAPI; 1:3 diluent, quarter-strength ringer’s solution) was added to obtain the resuspended bacterial culture. The cell samples were then placed onto a microslide and placed in the dark for 10 min. The fluorescence of DAPI in cells was observed using an inverted fluorescence microscope (Olympus IX71; Olympus, Tokyo, Japan). Each experiment was repeated three times (8).

### Enzyme preparation

DNA Topo I and TopoII were extracted from *S.aureus*. Topoisomerase activity was examined using the DNA relaxation reaction. One unit of topoisomerase activity was defined as the quantity of enzyme required to fully relax 0.5 μg supercoiled pBR322 DNA (9).

### Effects of fraxetin on DNA topoisomerase activity

DNA relaxation assays were based on the following procedure: 0.5 μg pBR322, 1 U of Topo I/II and 2 μl fraxetin (final concentrations 0.02, 0.05 and 0.08 mg/ml) or 100% ethanol (control) was added to 20 μl reaction buffer containing 10 mM Tris-hydrochloride (Tris-HCL, pH 7.5), 50 mM potassium chloride, 50 mM sodium chloride, 5 mM magnesium chloride, 0.1 mM EDTA, 15 mg/ml bovine serum albumin and 1 mM adenosine triphosphate (ATP; omitted in the Topo I-mediated DNA relaxation). The reaction was performed at 37°C for 30 min and inhibited by the addition of 1 μl Proteinase K (10 mg/ml) and 1 μl 10% sodium dodecyl sulfate (SDS) at 37°C for 30 min. The DNA samples were loaded onto a 1% agarose gel and visualized using a transilluminator (10,11).

### Effects of fraxetin on DNA

Various concentrations of fraxetin (final concentrations 0.01, 0.03 and 0.05 mg/ml) and 0.5 μg pBR322 were added to 2.5 μl helicase buffer I or helicase buffer II (as mentioned above), with the final reaction volume made up to 20 μl with distilled water. The reaction was incubated at 37°C for 30 min. Following incubation, 1 μl proteinase K (10 mg/ml) and 2 μl 10% SDS were added and incubated at 37°C for an additional 30 min to inhibit enzyme activity. Ethanol was used as a control group. The samples were loaded onto a 1% agarose gel and visualized using a transilluminator (12,13).

Fraxetin (final concentration 0.02 mg/ml) and pBR322 (final concentration 0, 0.25, 0.35, 0.45 and 0.55 mg/ml) were dissolved in 1.5 ml Tris-HCl (pH 7.2). Following incubation at 37°C for 30 min, the samples were examined using a UV-1100 ultraviolet spectrophotometer at 300–450 nm (14).

### Effects of fraxetin on DNA restriction enzyme digestion

Fraxetin (0.05 mg/ml) and pBR322 (1 μg) were dissolved in 0.5 μl Tris-HCl and incubated at 37°C for 30 min. The digestive reaction was performed using 1 μl *Taq*I, *Eco*RI, *Eco*RII, *Hin*dIII, *Bam*HI and *Sal*I, respectively and 2 μl loading buffer at 37°C for 30 min, with the final reaction volume made up to 20 μl. The samples were then loaded onto a 1% agarose gel and visualized using a transilluminator.

## Results

### Effect of fraxetin on the cell membrane of S. aureus

**Figure 1:**
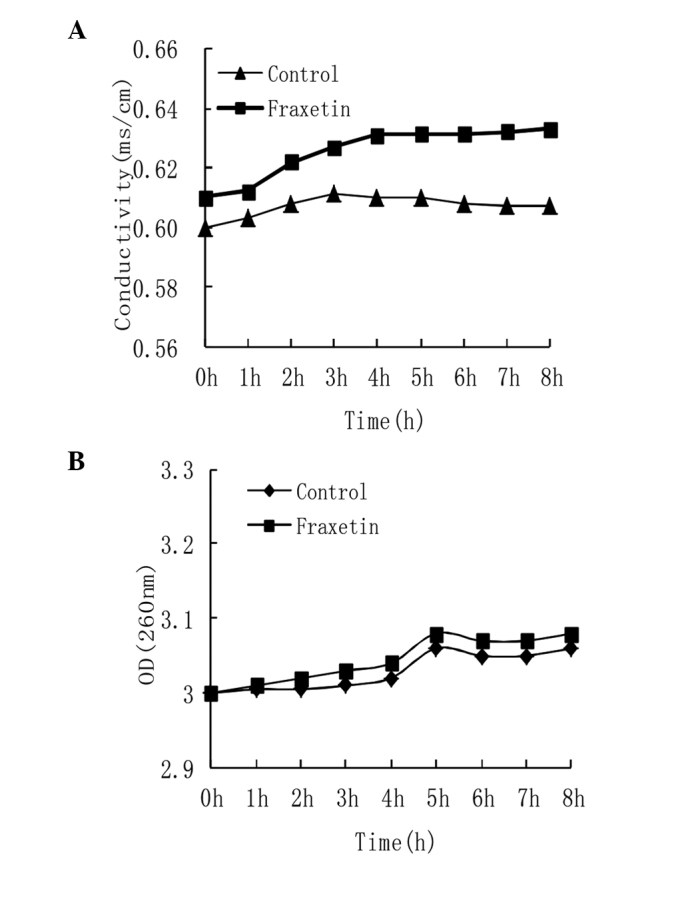
Changes in the (A) conductivity and (B) OD of macromolecules in *Staphyloccus aureus* following treatment with fraxetin. OD, optical density.

The cell membrane integrity can be conjectured by measuring the alterations in electrical conductivity and macromolecules, including DNA and RNA in culture medium following the addition of drugs. The results indicated that the electrical conductivity increased as the incubation time with fraxetin increased. Following treatment of *S. aureus* with fraxetin for 8 h, conductivity was increased by 5% (P<0.05), compared with the control group (Fig. 1A), which demonstrated that fraxetin had an effect on the integrity of the membrane.

The release of macromolecular material, including DNA and RNA can further demonstrate the effect of fraxetin on cell membrane integrity. The result revealed little change in the content of DNA and RNA in culture medium following treatment with fraxetin, compared with the control group (P>0.01; Fig. 1B), which demonstrated that the fraxetin did not disintegrate the cell membrane but did cause a small degree of damage.

### Effect of fraxetin on S. aureus soluble protein synthesis

**Figure 2:**
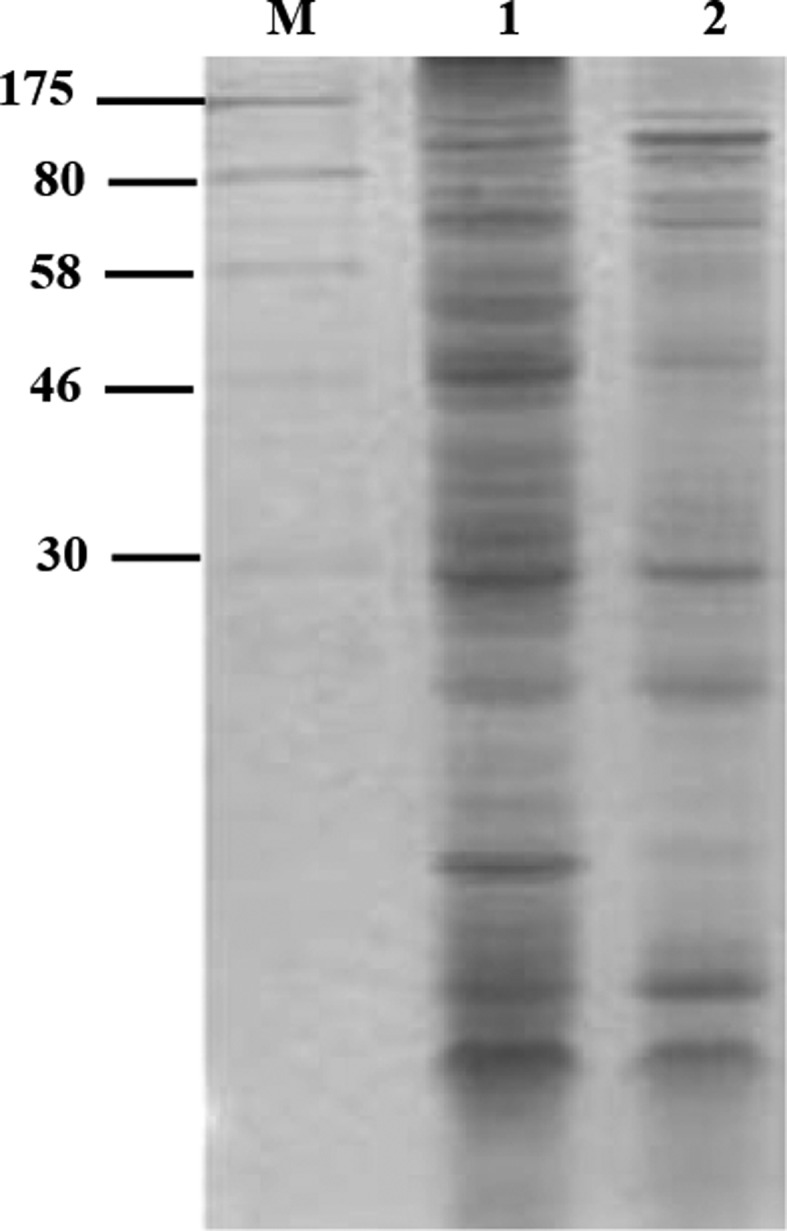
Changes in soluble protein in *Staphylococcus aureu*s following treatment with fraxetin. M, marker; 1, control group; 2, fraxetin 16 h group.

The result of SDS-PAGE indicated that soluble protein synthesis was significantly reduced by 55.74% (P<0.01) following treatment of *S. aureus* with fraxetin for 16 h (Fig. 2), compared with the control group. This may be due to fraxetin inhibiting nucleic acid synthesis and the expression of associated genes.

### Effect of fraxetin on S. aureus nucleic acid synthesis

**Figure 3:**
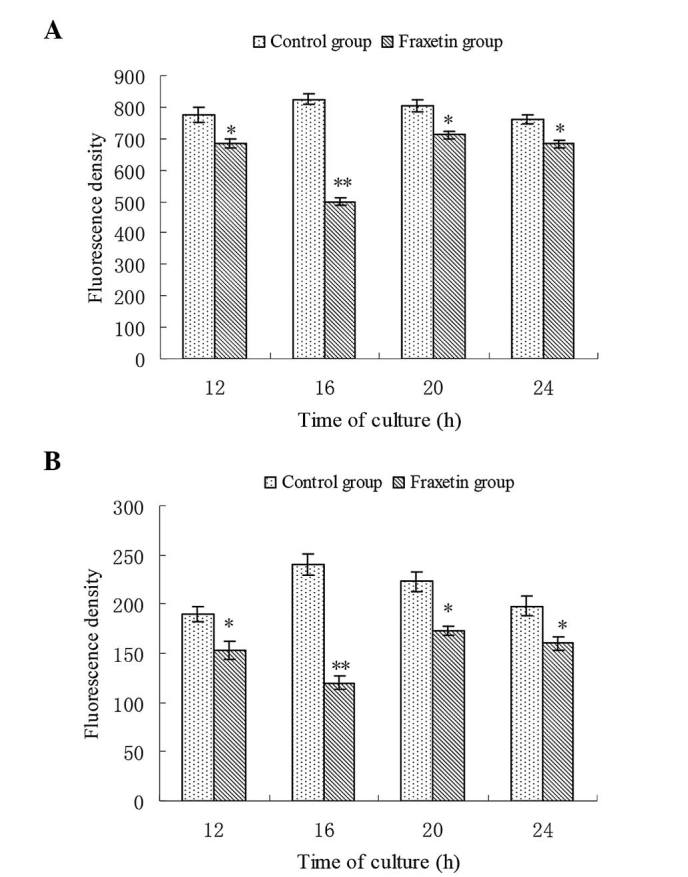
Effect of Fraxetin on the synthesis of nucleic acids in *Staphylococcus aureus* for 24 h by flourescence spectrometry. (A) Effect on DNA levels. (B) Effect on RNA levels. Error bars indicate the mean ± standard error, n=3, ^*^P<0.05, ^**^P<0.01 vs. control group.

DAPI is a fluoresecent dye that binds DNA and RNA. The dye increases in its fluorescence with increases in the quantity of nucleic acids. In the *S. aureus* cells treated with fraxetin for 16 h, the fluorescence spectrophotometry measurements demonstrated that DNA synthesis was significantly reduced up to 33.86% and that RNA synthesis was significantly reduced up to 48.96% compared with the control group, indicating that fraxetin had an adverse effect on nucleic acid synthesis. However, after 16 h, the nucleic acid content of the cells demonstrated an increasing trend, which may have been due to the increase in incubation time, the effectiveness of the drug gradually being reduced or the cells repairing the damaged DNA (Fig. 3).

### Effect of fraxetin on DNA topoisomerase activity

**Figure 4:**
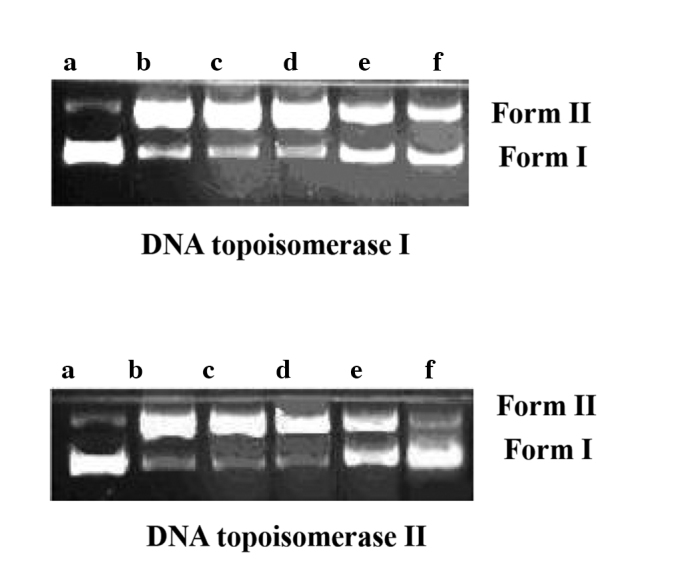
Effect of fraxetin on topoisomerase I and II in *Staphylococcus aureus*. (a) pBR322 DNA; (b) 1U topoisomerase; (c) absolute ethyl alcohol negative control; (d-f) 0.02, 0.05 and 0.08 mg/ml fraxetin. Form I, supercoiled DNA; Form II, open circular DNA and linear DNA.

The effect of fraxetin on the strand passage activity of topoisomerase was determined by enzyme-mediated negatively supercoiled pBR322 relaxation. As shown in Fig. 4, the activity of topoisomerase I and II was significantly inhibited from a fraxetin concentration of 0.05 mg/ml. These results suggested that fraxetin acted on topoisomerase I and II, thereby affecting nucleic acid synthesis and inhibiting bacterial growth.

### Interaction of fraxetin and DNA

**Figure 5:**
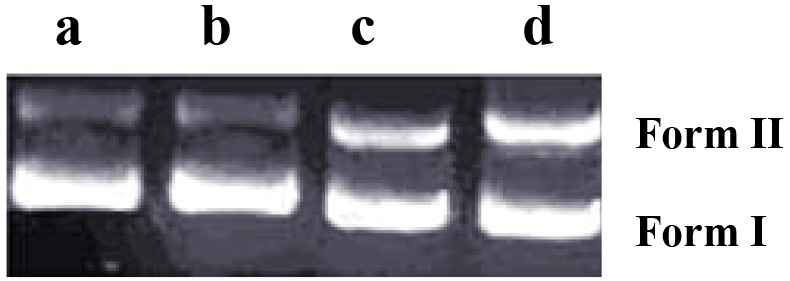
Direct break effect of fraxetin on pBR322 DNA. Form I, supercoiled DNA; Form II, open circular DNA and linear DNA.

**Figure 6:**
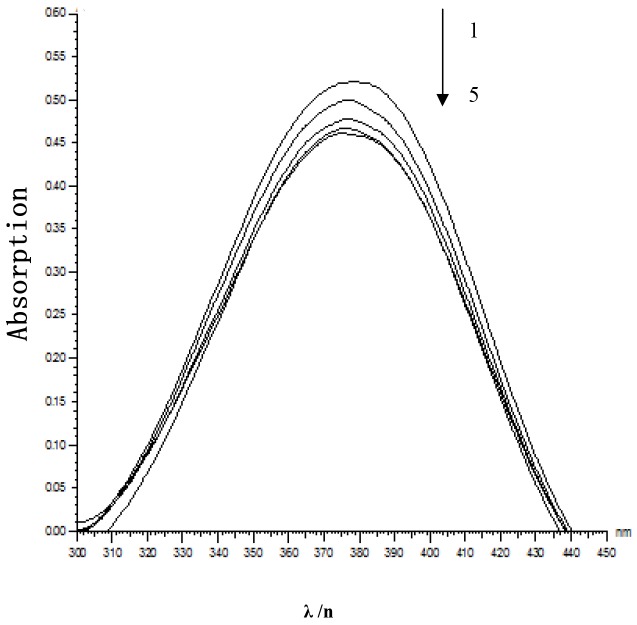
UV-Visible absorption spectra of the fraxetin-DNA system. C_Fraxetin_ = 0.02 mg/ml; C_DNA_ = 0, 0.25, 0.35, 0.45 and 0.55 mg/ml.

**Figure 7:**
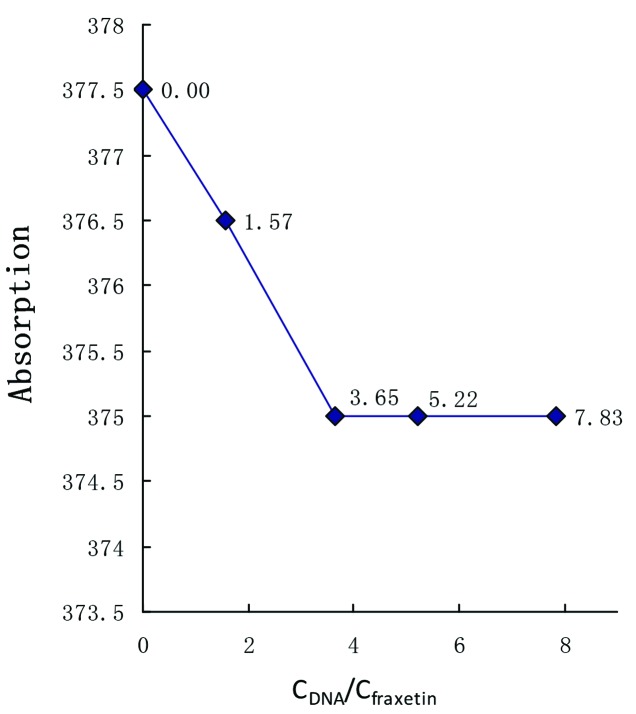
Association between fraxetin and C_DNA_/C_fraxetin_ in pH 7.0 Tris-hydrochloride buffer solution. C indicates concentration.

In order to investigate the direct cleavage effect of fraxetin on DNA, pBR322 DNA was incubated with different concentrations of fraxetin. With increasing concentrations of fraxetin, the quantity of supercoiled DNA (Form I) decreased, while open circular DNA and linear DNA (Form II) increased, indicating that fraxetin was able to interact with DNA and cleave it (Fig. 5). UV-visible spectrophotometric determinations of fraxetin demonstrated characteristic blue shift, hypochromism and isosbestic points with increases in DNA concentration (Fig. 6)*.* The results indicated that the binding parameters of fraxetin with DNA were 3.6±1 (Fig. 7).

### Effect of restriction enzymes

**Figure 8:**
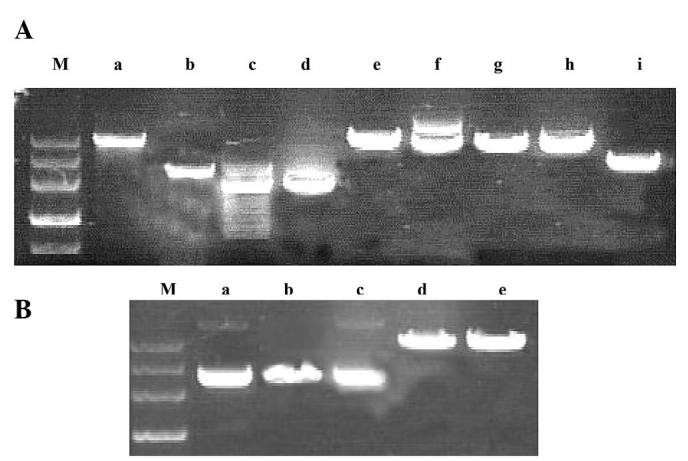
Effect of fraxetin on the macrorestriction map of restriction enzymes. (Aa) pBR322+Taq; (Ab) pBR322+*Taq*+fraxetin; (Ac) pBR322+*Eco*RII; (Ad) pBR322+*Eco*RII+fraxetin; (Ae) pBR322+*Hin*dIII; (Af) pBR322+*Hin*dIII+fraxetin; (Ag) pBR322+*Sal*I; (Ah) pBR322+*Sal*I+fraxetin; (Ai) pBR322. (Ba) pBR322; (Bb) pBR322+*Eco*RI; (Bc) pBR322+*Eco*RI+fraxetin; (Bd) pBR322+*Bam*HI; (Be) pBR322+*Bam*HI+fraxetin. M, marker.

The results mentioned above indicated that fraxetin interacted intercalatively with DNA. A total of six DNA restriction enzymes (*Taq*I, *Eco*RI, *Eco*RII, *Hin*dIII, *Sal*I and *Bam*HI) with different cut sites were used to predict the binding points between fraxetin and DNA through observing a digestion map (Fig. 8A and B). The observation indicated that fraxetin specifically inhibited the digestion of DNA by *Taq*I and *Hin*dIII, which recognizes the T/CGA and A/AGCT sites, and thus may be the result of fraxetin binding to the T/CGA, A/AGCT sites or a similar site.

## Discussion

The general mechanisms of antibacterial drugs include damaging the integrity of the cell wall and the cell membrane and inhibiting the synthesis of proteins and nucleic acids (15). The electrical conductivity results indicated that fraxetin did not cause destruction of the integrity of the cell wall or cell membrane and that the target of bacteriostasis was intracellular. Protein band quantitative analysis demonstrated that fraxetin affects protein synthesis, as the protein content of solution reduced by 55.74% (P<0.01) following treatment with fraxetin for 16 h. Incubating *S. aureus* with fraxetin for 16 h significantly reduced the quantities of DNA and RNA by 33.86 (P<0.01) and 48.96% (P<0.01), respectively, compared with the control group. This confirmed that there was a change in the genetic material, which may explain the change in protein content. The results from our previous experiments indicated that the change in nucleic acid content was associated with the drug inhibiting DNA topoisomerase (16). DNA topoisomerase is necessary for DNA replication, facilitating the short-term separation of single-stranded or double-stranded DNA (17,18). The results demonstrated that fraxetin significantly inhibited the activity of DNA topoisomerase I and II, with inhibition of topoisomerase I and II activity at 0.05 mg/ml fraxetin and complete inhibition at 0.08 mg/ml fraxetin. Therefore, from this observation, it was inferred that DNA topoisomerase could be one of the direct targets for the antibacterial action of fraxetin. Previous studies have demonstrated that the mechanism of topoisomerase inhibitors was to produce drug-topoisomerase-DNA cleavable complexes or to interfere with the binding of topoisomerase to DNA (19,20). Results from the present study revealed that the UV-visible absorption spectrum altered as the concentration of DNA changed and exhibited hypochromism and a blue shift. According to the criteria proposed by Long and Barton (14), through the observation of hypochromism and blue shifts following treatment of small molecules with DNA, it can be inferred that the drug had an intercalative interaction with DNA. Therefore, the change in absorption characteristics was due to the electronic interaction between the intercalator and the DNA bases.

In the present study, different DNA restriction enzymes were used and the digestion maps were observed to determine the binding sites following treatment with fraxetin (19). Among the four tests, fraxetin specifically inhibited the digestion of DNA by *Taq*I and *Hin*dIII, which recognize the T/CGA and A/AGCT sites. This was due to the drug binding to the T/CGA, A/AGCT or similar sites, however, the specific mechanism of interaction requires further investigation.

In conclusion, it was hypothesized that the inhibitory mechanism of fraxetin on *S. aureus* may be associated with the intercalative interaction between the drug and DNA. This results in the loss of topoisomerase activity and has effects on the replication and transcription of DNA and the synthesis of proteins, which in turn prevents the division of bacterial cells.
